# The lebercilin-like protein is embedded in a ciliary protein network and is preferentially expressed in motile cilia

**DOI:** 10.1186/2046-2530-1-S1-P93

**Published:** 2012-11-16

**Authors:** DA Mans, KLM Coene, K Boldt, IJC Lamers, J van Reeuwijk, NT Loges, E Bolat, L Franke, L Hetterschijt, SJF Letteboer, TA Peters, H Omran, FPM Cremers, M Ueffing, R Roepman

**Affiliations:** 1 Department of Human Genetics, Nijmegen Centre for Molecular Life Sciences, Radboud University Nijmegen Medical Centre, the Netherlands; 2 Division of Experimental Ophthalmology and Medical Proteome Center, Center of Ophthalmology, University of Tübingen, Germany; 3 Children's Department, University Hospital Münster, Germany; 4 Department of Genetics, University Medical Center Groningen and University of Groningen, Germany; 5 Department of Otorhinolaryngology, Nijmegen Centre for Molecular Life Sciences, Radboud University Nijmegen Medical Centre, the Netherlands; 6 Donders Institute for Brain, Cognition and Behaviour, the Netherlands; 7 Department of Protein Science, Helmholtz Zentrum München, German Research Center for Environmental Health, Germany

## 

Mutations in *LCA5* are causative for Leber congenital amaurosis, a severe hereditary retinal dystrophy in humans. Lebercilin, encoded by *LCA5*, localizes to connecting cilia of photoreceptor cells in the retina and specifically interacts with the intraflagellar transport (IFT) machinery. Bioinformatic analysis has identified lebercilin-like protein, previously known as C21orf13, as a lebercilin homolog in humans. In this study, we have characterized the molecular properties of lebercilin-like protein by defining the lebercilin-like interactome and assessing its (sub)cellular localization in ciliated cells. We show that lebercilin-like protein is embedded in a ciliary protein network and specifically localizes at the basal body and ciliary axoneme of ciliated cells, like lebercilin. mRNA expression studies indicate that lebercilin-like protein is preferentially expressed in tissues featuring motile cilia and/or flagella. Based on these data and bioinformatic co-expression profiling, we suggest that *LCA5L* is a likely candidate gene for motile ciliopathies such as Primary Ciliary Dyskinesia (PCD).

